# A novel RBD-protein/peptide vaccine elicits broadly neutralizing antibodies and protects mice and macaques against SARS-CoV-2

**DOI:** 10.1080/22221751.2022.2140608

**Published:** 2022-11-11

**Authors:** Shixia Wang, Chang Yi Wang, Hui-Kai Kuo, Wen-Jiun Peng, Juin-Hua Huang, Be-Shen Kuo, Feng Lin, Yaw-Jen Liu, Zhi Liu, Huang-Ting Wu, Shuang Ding, Kou-Liang Hou, Jennifer Cheng, Ya-Ting Yang, Ming-Han Jiang, Min-Sheng Wang, Tony Chen, Wei Guo Xia, Ed Lin, Chung Ho Hung, Hui-Jung Chen, Zhonghao Shih, Yi Ling Lin, Valorie Ryan, Mei Mei Hu, D. Gray Heppner, Delphine C. Malherbe, Sivakumar Periasamy, Natalia Kuzmina, Chandru Subramani, Michael Hellerstein, Thomas P. Monath, Alexander Rumyantsev, Alexander Bukreyev, Farshad Guirakhoo

**Affiliations:** a Vaxxinity, Inc., Dallas, TX, USA; b United Biomedical Inc., Asia, Taipei, Taiwan; c United BioPharma, HuKo, Taiwan; d United Biomedical Inc., Hauppauge, NY, USA; e Emerging Infectious Disease Division, Academia Sinica, Nangang, Taiwan; f Department of Pathology, University of Texas Medical Branch, Galveston, TX, USA; g Department of Microbiology & Immunology, University of Texas Medical Branch, Galveston, TX, USA; h Galveston National Laboratory, Galveston, TX, USA

**Keywords:** SARS-CoV-2, subunit vaccine, neutralizing antibody, protection, non-human primate

## Abstract

The development of safe and effective vaccines to respond to COVID-19 pandemic/endemic remains a priority. We developed a novel subunit protein-peptide COVID-19 vaccine candidate (UB-612) composed of: (i) receptor binding domain of SARS-CoV-2 spike protein fused to a modified single-chain human IgG1 Fc; (ii) five synthetic peptides incorporating conserved helper and cytotoxic T lymphocyte (Th/CTL) epitopes derived from SARS-CoV-2 structural proteins (three from S2 subunit, one from membrane and one from nucleocapsid), and one universal Th peptide; (iii) aluminum phosphate as adjuvant. The immunogenicity and protective immunity induced by UB-612 vaccine were evaluated in four animal models: Sprague–Dawley rats, AAV-hACE2 transduced BALB/c mice, rhesus and cynomolgus macaques. UB-612 vaccine induced high levels of neutralizing antibody and T-cell responses, in all animals. The immune sera from vaccinated animals neutralized the SARS-CoV-2 original wild-type strains and multiple variants of concern, including Delta and Omicron. The vaccination significantly reduced viral loads, lung pathology scores, and disease progression after intranasal and intratracheal challenge with SARS-CoV-2 in mice, rhesus and cynomolgus macaques. UB-612 has been tested in primary regimens in Phase 1 and Phase 2 clinical studies and is currently being evaluated in a global pivotal Phase 3 clinical study as a single dose heterologous booster.

## Introduction

The COVID-19 pandemic remains a significant concern driven by the continued emergence of variants of concern (VOCs) and the short duration of protection from natural infection or vaccination. The most recent VOC, Omicron (B.1.1.529) and its sub-lineages (BA.1, BA.2, BA.4, BA.5 and BA.2.12.1) have quickly spread globally breaking through the immune protection generated by vaccines and/or natural infections [1–4]. In response to this unprecedented public health crisis, many vaccine platforms, including inactivated whole virus, subunit recombinant proteins, recombinant adenovirus-based vectors, and nucleic acid approaches, have been used to develop 40 commercial vaccines authorized as of July 2022 [5].

Most authorized COVID-19 vaccines were designed to elicit binding and neutralizing antibodies (NAbs) using full-length spike (S) protein or receptor binding domain (RBD) as immunogen. The binding and NAb responses are strongly correlated with protection against SARS-CoV-2 infection, clinical manifestations, and disease progression in COVID-19 [6]. The RBD contains key epitopes for inducing NAb responses; therefore, it is one of the major targets for developing future vaccines against SARS-CoV-2 and related sarbecoviruses. It was demonstrated that over 90% of SARS-CoV-2 NAbs in convalescent sera or vaccinated subjects are directed to RBD [7]. The RBD-based vaccines offer high-yielding, cost-effective manufacturing productions (e.g. using mammalian cell, bacterial, yeast and plant cell expression systems) and temperature stability addressing equitable global access to COVID-19 vaccines especially in low-and-middle-income countries [8]. Currently, multiple RBD-based vaccines are in clinical development, and some have shown favorable clinical efficacy against COVID-19 [9–11].

In UB-612 vaccine composition, a Chinese Hamster Ovary (CHO)-cell-produced RBD fused to a modified single-chain human IgG1 Fc (sFc) protein (RBD-sFc, S1-RBD-sFc) serves as the immunogen to generate humoral responses. UB-612 vaccine also contains five promiscuous synthetic S2/M/N peptides composed of helper and cytotoxic T-cell (Th/CTL) epitopes derived from S2 subunit of S protein (3 peptides), membrane (M, 1 peptide), and nucleocapsid (N, 1 peptide) to induce cross-reactive cell-mediated immune responses against SARS-CoV-2; and one proprietary Th peptide, derived/modified from measles virus fusion protein to catalyze T-cell activation [12]. In UB-612 vaccine formulation, synthetic peptides are stabilized with a proprietary oligonucleotide (CpG1) in a complex by charge interaction and adsorbed on aluminum phosphate (Adju-Phos, an adjuvant used in humans for over 70 years).

This report evaluated the immunogenicity and protective efficacy of the UB-612 vaccine candidate against SARS-CoV-2 infection in four animal models, including rats, adeno-associated virus (AAV) transduced mice expressing human angiotensin-converting enzyme 2 (AAV/hACE2), rhesus and cynomolgus macaques.

## Materials and methods

### UB-612 vaccine components

The UB-612 vaccine formulation (Supplemental Figure S1) is comprised of RBD-sFc protein and five conserved Th/CTL S2/M/N peptides as immunogen. RBD-sFc protein was produced from a stable CHO cell line. To identify the desirable CTL T-cell epitopes in SARS-CoV-2 S, M and N proteins, we employed “Epitope Prediction and Analysis Tools” [13, 14]. CpG1 is a 32-mer type B oligodeoxynucleotide sequence [15]. It is used as an excipient (at 4 μg/mL, which is well-below its adjuvant activity at 0.5–3 mg per dose used in various vaccines) to bind the positively charged Th/CTL peptides (by design) by dipolar interactions [13, 16]. The RBD-sFc and peptide production details are described in Supplemental Materials.

To prepare UB-612 vaccine for animal studies, RBD-sFc protein was first formulated with UBITh®1a Th peptide and adsorbed on Adju-Phos adjuvant (InvivoGen) at 1.6 μg/mL, before mixing with five S2/M/N T-cell peptides (4 μg/mL each) in CpG1 excipient (4 μg/mL). In UB-612 formulation, the weight:weight ratio of immunogens is 88% of RBD-sFc protein and 12% of T-cell peptides in each dose for rat (Supplemental Materials), mouse and macaque immunization studies.

### Mouse immunization and challenge study

The immunogenicity and protective immunity of UB-612 vaccine were evaluated in an AAV/hACE2 transduced BALB/c mouse model [17]. Four groups (n = 4) of 8-week-old male BALB/c mice were administered with 3, 9 or 30 μg of UB-612 vaccine or saline by intramuscular injection (IM) at Weeks 0 and 2. Mouse sera were collected at Weeks 0, 3 and 4 to evaluate the RBD-specific IgG and NAb responses. Two weeks after the 2^nd^ immunization, mice were transduced by intratracheal (IT) inoculation with 3 × 10^11^ vector genomes (vg) of AAV6/hACE2. To transduce extrapulmonary organs, 1 × 10^12^ vg of AAV9/hACE2 was injected intraperitoneally [17]. Two weeks after transduction, the AAV6/CB-hACE2 mice were challenged with 10^6^ PFU (100 μL) of SARS-CoV-2 hCoV-19/Taiwan/4/2020 strain by intranasal (IN) inoculation. Mice were weighed daily for four days, and lung tissues were collected on Day 5 post-challenge for pathology evaluation. Mouse challenge experiments were performed in ABSL-3 facility. All mouse procedures were approved by institutional animal care and use committee (IACUC) at UBIAsia and Academia Sinica in Taiwan.

### Rhesus macaque immunization and challenge study (3-dose regimen)

We conducted two non-human primate (NHP) immunogenicity and protection studies in rhesus macaques (3-dose) and cynomolgus macaques (2-dose), respectively.

In rhesus macaques study similar to a previously established COVID-19 NHP model [18, 19], four groups (n = 4) of macaques (3-6 year-old, male/female) were immunized with 10, 30 or 100 μg of UB-612 vaccine or saline on Days 0, 28 and 70 at JOINN Laboratories (Beijing, China). Sera were collected on Days 0, 14, 28, 35, 42, 70 and 77 to evaluate RBD-specific IgG and NAb responses. Macaques were challenged on Day 81 (11 days after the 3^rd^ immunization) with 10^6^ 50% Tissue Culture Infectious Dose (TCID_50_) of SARS-CoV-2 WH-09/human/2020/CHN (WT) strain by IT at ABSL-3 facility. Virus loads in lungs were evaluated on Day 7 post-challenge using RT–PCR, expressed in viral RNA genome copies/gram of lung tissue. All procedures were approved by JOINN’s IACUC.

### Cynomolgus macaque immunization and challenge study (2-dose regimen)

Three groups (n = 5) of cynomolgus macaques (3–6 year-old, male/female) were immunized with 30 or 100 μg of UB-612 vaccine or saline on Days 0 and 28 at Biomere (Worcester, MA). The immunized animals were transferred to BIOQUAL’s ABSL-3 facility (Rockville, MD) for challenge study. Sera and peripheral blood mononuclear cell (PBMCs) were collected on Days 0, 14, 28 and 50 to evaluate RBD-specific IgG, NAb and cell-mediated immune responses. Four weeks after the 2^nd^ immunization (Day 55), animals were challenged by both intratracheal and intranasal routes with a total of 1.0 × 10^5^ TCID_50_ of SARS-CoV-2 USA/WA1/2020 (WA) strain, which was ∼5-fold higher dose than that used in a similar cynomolgus macaque challenge study at BIOQUAL for the authorized COVID-vaccine NVX-CoV2373 [20], to evaluate the UB-612 induced protection against more stringent high viral dose infection. The challenge dose was divided equally between IN (5 × 10^4^ TCID_50_) and IT (5 × 10^4^ TCID_50_) administrations. On Days 0, 3, 5, and 8 post-challenge, bronchoalveolar lavage (BAL) and nasal swabs were collected to assess viral shedding by subgenomic messenger RNA (sgmRNA) RT–PCR. All procedures were approved by Biomere and BIOQUAL IACUCs.

### Antibody assessments

RBD-specific IgG and RBD:hACE-2 binding inhibition antibody responses in mice, rats and macaques were evaluated by ELISA. Three types of SARS-CoV-2 neutralization assays were conducted to evaluate the NAb responses: live virus microneutralization, live virus cytopathic effect (CPE), and pseudovirus neutralization assays. Live virus microneutralization assays were performed using original SARS-CoV-2 WT and WA strains, Delta and Omicron BA.1 variants. The live virus CPE neutralization assays were performed using D614G, Alpha (B.1.1.7), Gamma (P.1), Beta (B.1.351), and Delta (B.1.617.v2) variants. Pseudovirus neutralizations were conducted using Vesicular stomatitis virus (VSV)-based pseudoviruses expressing S protein from WT strain, three VOCs: Alpha (B.1.1.7), Beta (B.1.351) and Gamma (P.1), two Variants of Interest (VOIs) Epsilon (B.1.429) and Iota (B.1.526). All live virus neutralization assays were performed in BSL-3 laboratory. The detailed methods are described in Supplemental Materials.

### Macaque T-cell response assessments

The S2/M/N peptide specific IFN-γ and IL-4 T-cell responses in cynomolgus macaques were evaluated in PBMCs collected at 3 weeks after the 2^nd^ immunization by T-cell ELISPOT and intracellular cytokine staining (ICS). PBMCs were cultured and stimulated in vitro with mixed S2/M/N peptides described previously [13] and in Supplemental Materials.

### Viral load detections

The viral loads were detected as virus TCID_50_ in mouse lungs and N gene sgmRNA levels in BAL fluid and nasal swabs of macaques, post-challenge. The detailed virus TCID_50_ and sgmRNA RT–PCR detection methods are described in Supplemental Materials.

### Lung histopathology

At the end of mouse and macaque challenge studies, animals were euthanized, and lung tissues were collected, trimmed, processed, embedded, sectioned, and stained with Hematoxylin and Eosin. Histopathology was examined under microscope and the histopathology scores were determined by microscopic examination (lung histopathology scoring details in Supplemental Materials).

### Statistical analysis

The results and analyses are descriptive. The data presented in the graphs are group geometric mean titers/values (GMT) with standard deviation (SD). An unpaired t-test or one-way ANOVA was performed for statistical analyses using GraphPad Prism. *P*-values *<0.05* were considered as significant differences.

## Results

### UB-612 vaccine

The UB-612 vaccine contains RBD-sFc and conserved T-cell epitopes derived from S2, M and N proteins (Supplemental Figure S1) [13, 14]. RBD-sFc (431 amino acids) protein, is the RBD (aa340-539 of S protein) region fused with the single-chain human IgG1 Fc at C-terminus. The IgG1 sFc was engineered with mutations (C220S, C226S, C229S, and N297H) eliminating two disulfide bonds and one N-linked glycan to disrupt the Fc effector functions. The mutations were expected to reduce a remote risk of possible depletion of effector cells carrying hACE2 receptors by C1q mediated killing [21]. Consequently, no antibodies could be detected against the Fc portion of RBD-sFc protein in animals and participants vaccinated with UB-612 in a Phase 1 clinical trial [13]. The RBD-sFc immunogen was selected based on screening of multiple RBD designs that generated highest binding and neutralizing antibody responses in guinea pigs (data not shown).

Synthetic T-cell peptides in UB-612 include three S2 peptides (p5752, aa957-984; p5753, aa891-917; and p5755, aa996-1028), one N peptide (p5754, aa305-331), and one M peptide (p5815, aa89-111), containing Th and/or CTL epitopes with known MHC-I and/or MHC-II binding and good manufacturability characteristics [13]. Each peptide was further modified by addition of a Lys-Lys-Lys tail at N-terminus to improve peptide solubility and enrich positive charge for interaction with negatively charged CpG1 excipient in vaccine formulation [22]. These five peptides are highly conserved in all SARS-CoV-2 variants to date. One S2 peptide (p5752) has 96% homology with Omicron variants (BA.1, BA.2, BA.2.12.1, BA.4 and BA.5), while the other four peptides from S2, M and N are 100% identical to Omicron variants. All five peptides have 100% homology with Delta variant.

The UB-612 vaccine was first demonstrated that it could elicit high levels of RBD-specific IgG, neutralizing antibody and T-cell responses in rats (Supplemental Materials).

### Protective immunity in AAV-hACE2 transduced mice

**Figure 1.**
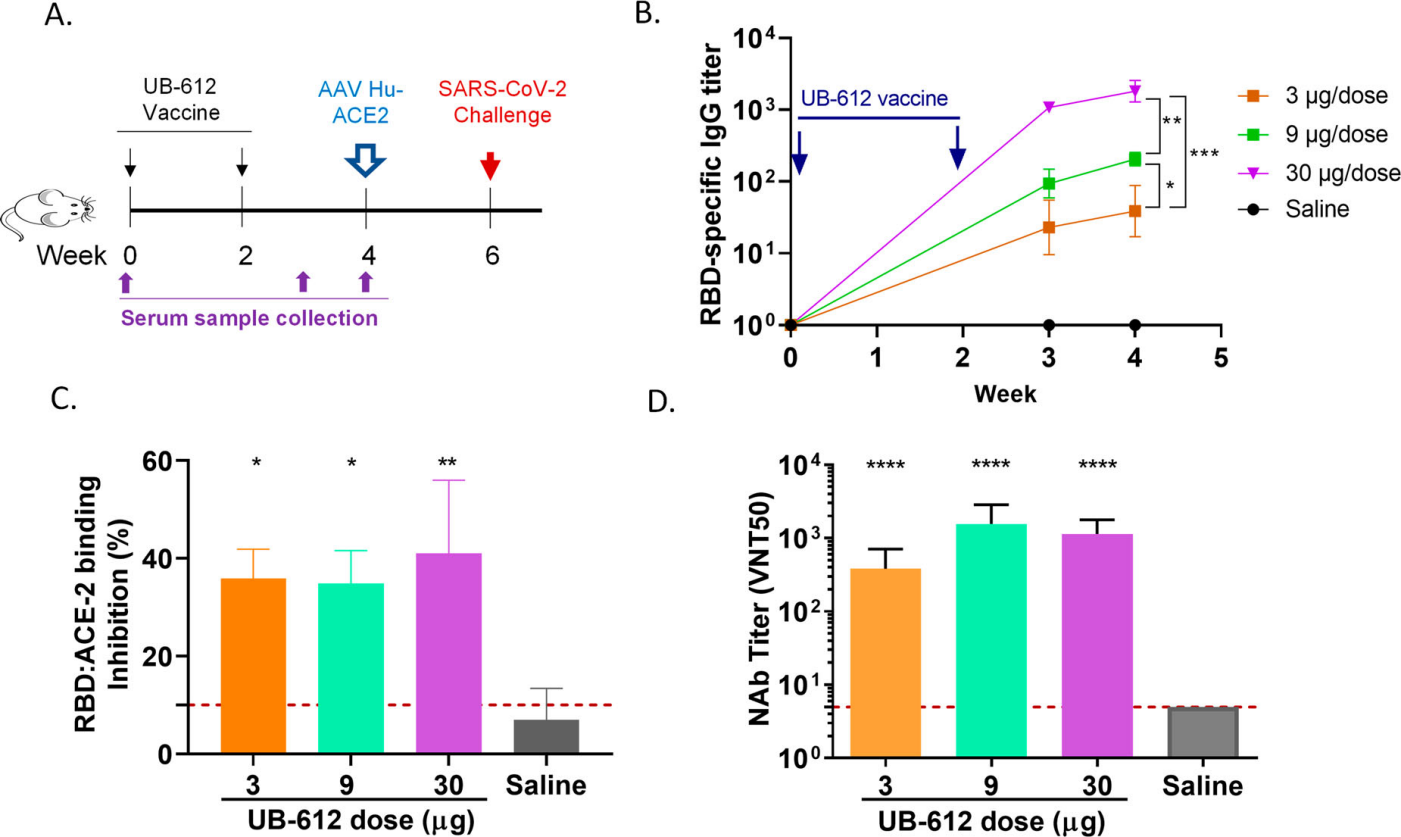
Antibody responses in UB-612 immunized BALB/C mice. (A) Immunization and challenge schedule. (B) Temporal RBD-specific antibody titers measured by ELISA. Arrows indicate immunization timepoints. Statistical significance is indicated as * *p < 0.05* between 3 and 9 µg groups; ** *p < 0.01* between 9 and 30 µg groups; *** *p < 0.005* between 3 and 30 µg groups. (C) RBD:hACE-2 binding inhibition titers at 2 weeks after the 2^nd^ immunization. Each bar represents GMT ± SD NAb titers with standard deviation. The red dash line indicates the assay cut-off. Statistical significance is indicated as * *p < 0.05*; ** *p < 0.01* compared to Saline group. (D) NAb titers against SARS-CoV-2 WT virus at 2 weeks after the 2^nd^ immunization. Statistical significance is indicated as ****p < 0.0001 between each vaccine dose and Saline group.

After two immunizations with UB-612 vaccine in mice, the RBD-specific IgG, RBD:hACE-2 binding blocking and neutralizing antibodies against a wild-type live virus hCoV-19/Taiwan/4/2020, were induced in all three dose groups (3, 9 or 30 µg) with a dose-dependent manner (Figure 1).

**Figure 2.**
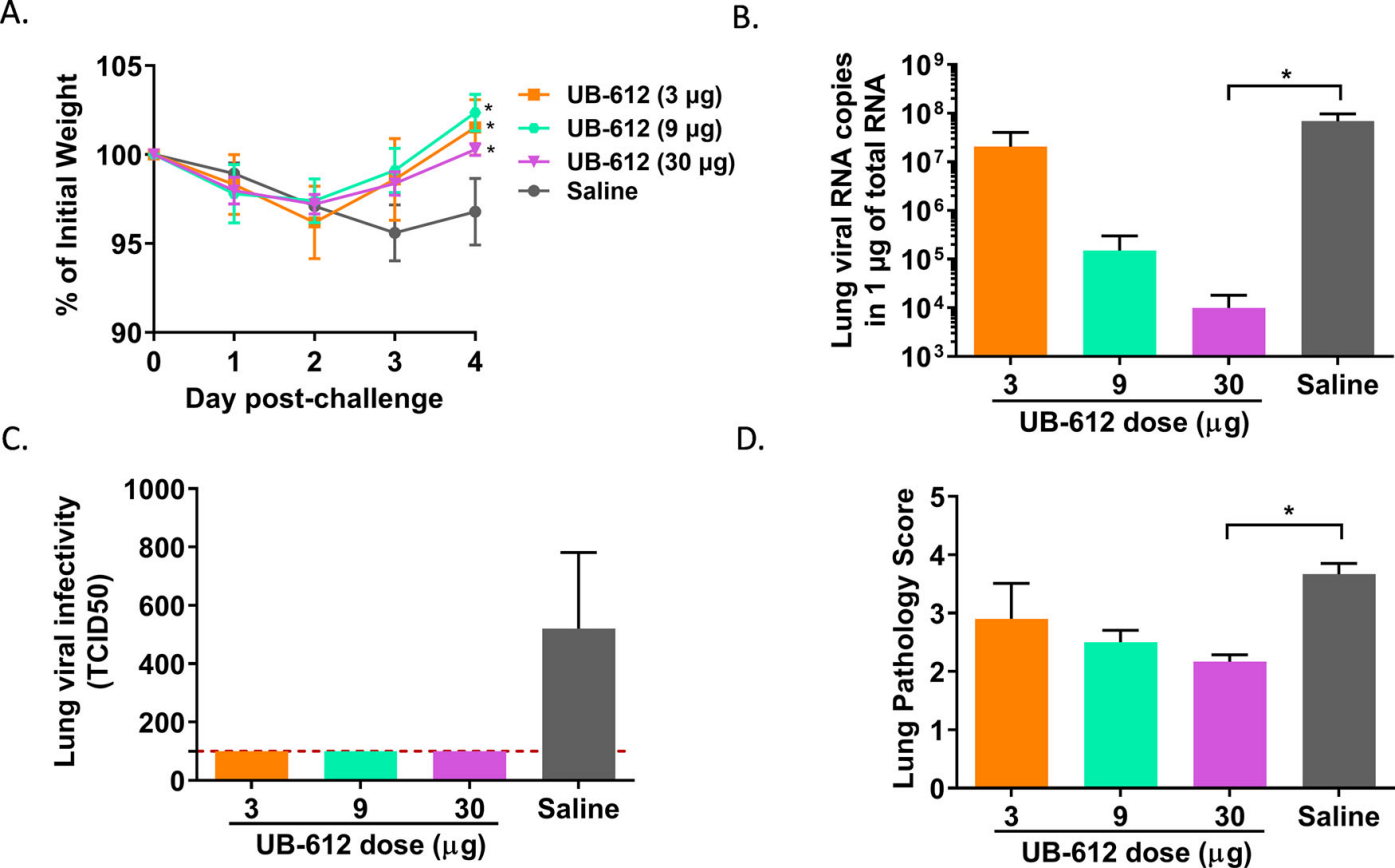
Protective immunity against SARS-CoV-2 challenge in AAV/hACE-transduced BALB/c mice. (A) Temporal body weight changes (%) to the baseline prior challenge. Each curve represents a group geometric mean with standard deviation. The statistical significance is indicated as * *p < 0.05*, each vaccine group compared to Saline group. (B) Lung viral RNA titers . (C) Lung virus TCID_50_. (D) Lung pathology scores. Each bar represents GMT ± SD on Day 5 post-challenge in panels B, C and D. The red dash line indicates the assay cut-off. Statistical significance is indicated as * *p < 0.05*; ** *p < 0.01* compared to Saline group.

Two weeks post the 2^nd^ immunization, mice were transduced with a recombinant AAV/hACE2. Two weeks later, the transduced mice were intranasally challenged with 10^6^ TCID_50_ of SARS-CoV-2 strain hCoV-19/Taiwan/4/2020 (Figure 1(A)). The vaccine efficacy was determined by body weight loss, lung viral load, and lung pathology score (Figure 2). Mice in 3 vaccine groups had significant lower levels of body weight loss compared to saline group (Figure 2(A)). Vaccination with 9 or 30 μg of UB-612 significantly reduced lung viral loads by ∼2.0 and 3.5 logs compared to saline group, respectively (Figure 2(B)). Infectious viral TCID_50_ titers in lungs were also significantly reduced in three vaccine groups (Figure 2(C)). The lung pathology demonstrated that the high dose (30μg) group had significant pathological score reduction compared to saline group (Figure 2(D), Supplemental Figure S4).

### Immunogenicity and protection against SARS-CoV-2 challenge after three doses of UB-612 vaccine in rhesus macaques

#### Antibody responses

**Figure 3.**
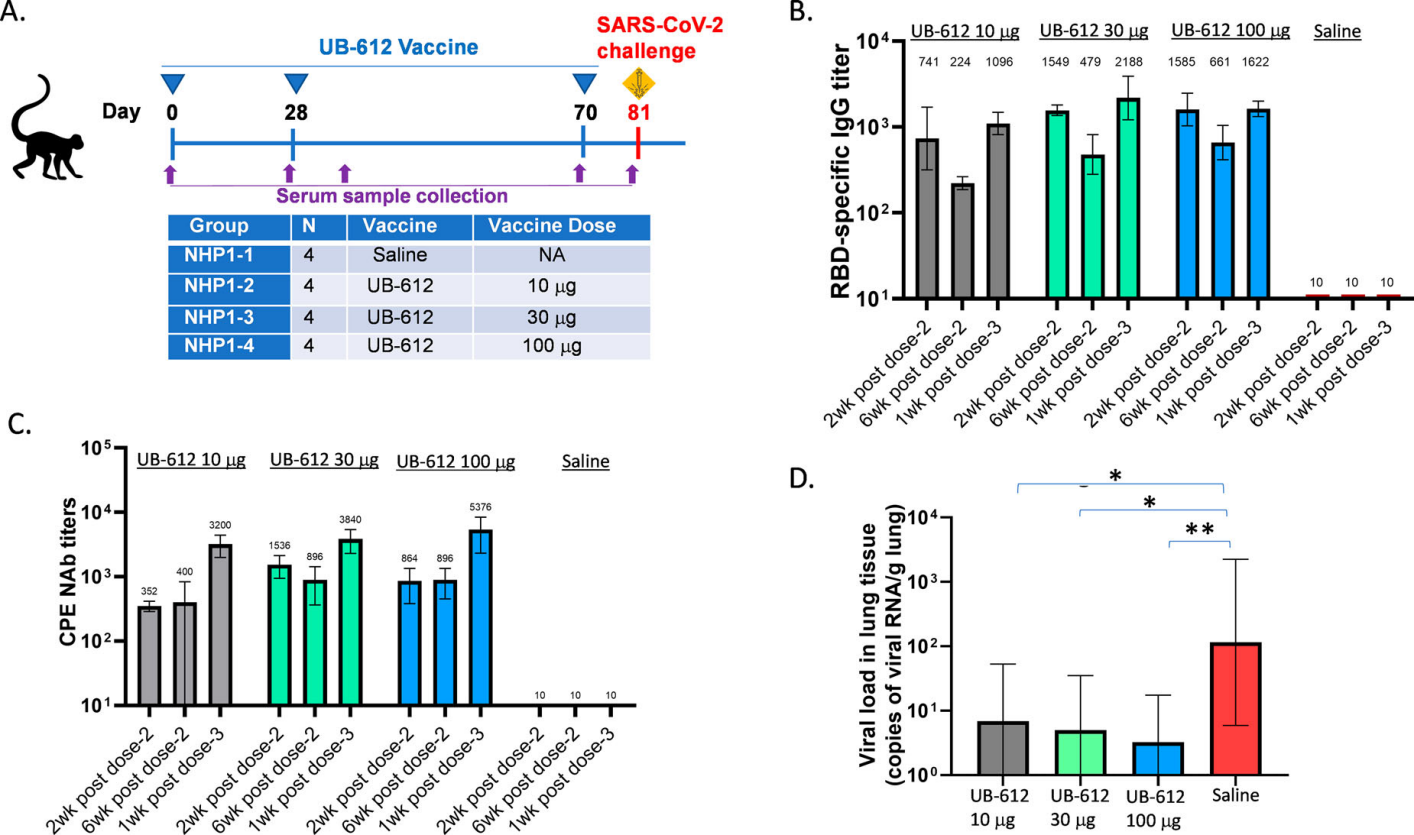
Antibody responses and protection in rhesus macaques. (A) Vaccine groups, dosing, and challenge schedule. (B) RBD-specific antibody responses at 2 or 6 weeks after the 2^nd^ immunization and 1 week after the 3^rd^ immunization. (C) CPE NAb titers against SARS-CoV-2 WT strain at 2 or 6 weeks after the 2^nd^ immunization and 1 week after the 3^rd^ immunization. (D) Lung viral RNA levels at 7 days post-challenge. Each bar represents GMT ± SD NAb titers or viral RNA loads. Statistical significance is indicated as * *p < 0.05*; ** *p < 0.01* compared to Saline group.

In the first NHP study, four groups of rhesus macaques were given 10, 30 or 100 μg of UB-612 or saline on Days 0, 28, and 70 as prime-boost immunization (Figure 3(A)). After two immunizations, all three vaccine dose groups elicited RBD-specific IgG and NAb responses. The RBD-specific IgG titers reached peak at two weeks post the 2^nd^ dose (Day 42) and started to wane six weeks post the 2^nd^ immunization (Day 70) (Figure 3(B)). The NAb titers were similar at two or six weeks post the 2^nd^ dose (Figure 3(C)), before the 3^rd^ booster dose on Day 70. One week after the 3^rd^ booster dose, RBD-specific IgG titers were increased by 2∼5-folds (Figure 3(B)). The NAb titers against SARS-CoV-2 WT strain were increased by >5-fold compared to pre-boost titers in all three dose groups (Figure 3(C)). The saline group showed no antibody responses, as expected.

#### Protection against SARS-CoV-2 WT strain challenge

Eleven days after the 3^rd^ booster dose (Day 81), the rhesus macaques were challenged by IT route with SARS-CoV-2 WT strain (10^6^ TCID_50_). In UB-612 vaccine groups (10, 30 or 100 μg), viral RNA loads in lungs were significantly reduced compared to saline group (Figure 3(D)). There was a trend of dose-dependent protection.

#### Neutralization against multiple VOCs

**Figure 4.**
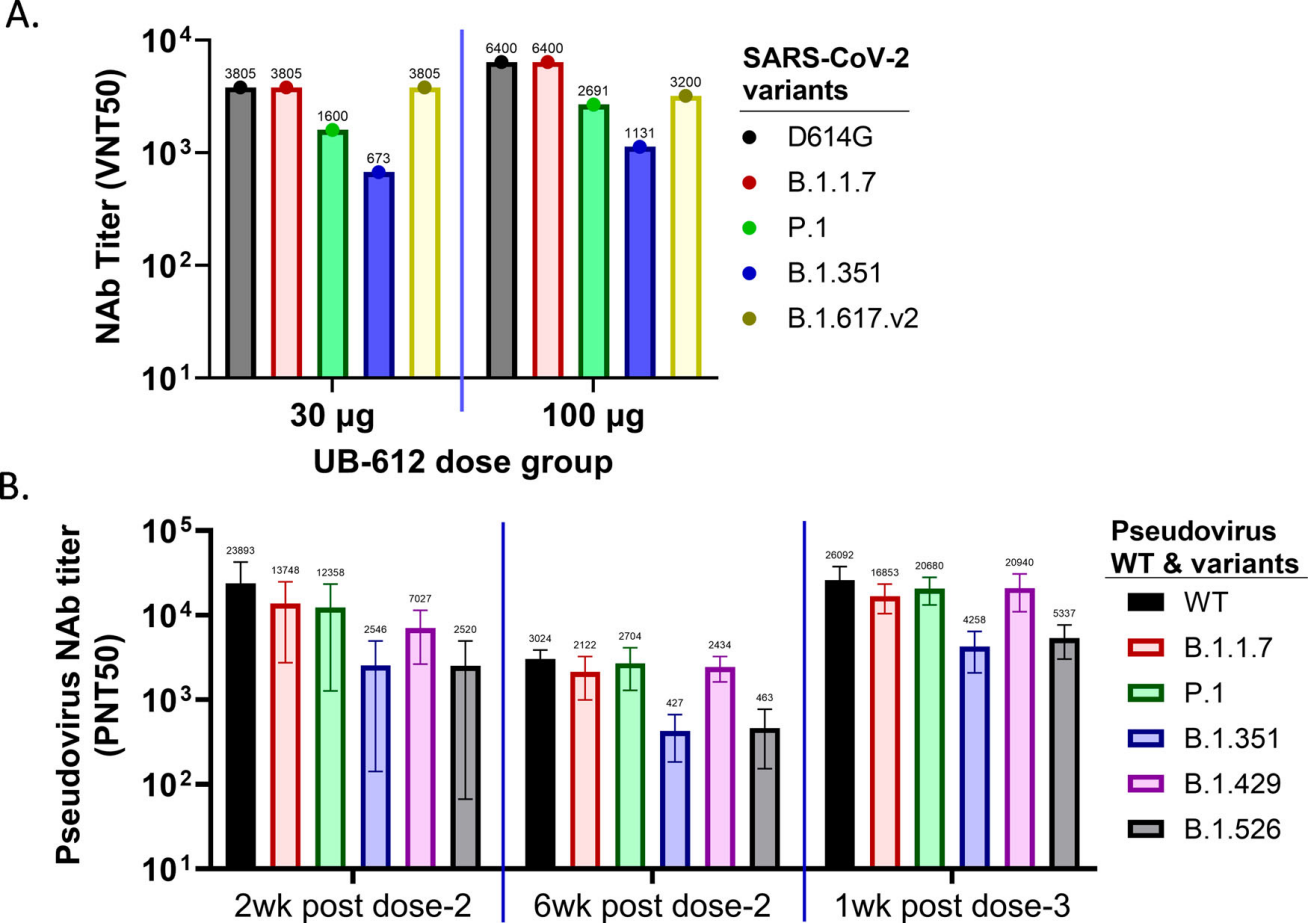
Serum NAb responses against SARS-CoV-2 VOCs and VOIs in UB-612 immunized rhesus macaques. (A) CPE NAb titers in group-pooled macaque sera against live viruses: D614G, Alpha (B.1.1.7), Beta (B.1.351), Gamma (P.1) and Delta (B.1.617.v2) in 30 and 100 μg dose groups at 1 week after the 3^rd^ immunization. The numbers on top of each bar represent the NAb titer in group-pooled sera. (B) NAb titers against SARS-CoV-2 pseudoviruses expressing S protein from original Wuhan strain (WT), 3 VOCs: Alpha (B.1.1.7), Beta (B.1.351) and Gamma (P.1), and 2 VOIs: Epsilon (B.1.429) and Iota (B.1.526). Sera were collected at 2 or 6 weeks after the 2^nd^ immunization, and 1 week after the 3^rd^ immunization. Each bar represents GMT ± SD NAb titer, with GMT value above.

The rhesus macaque sera collected at one week after the 3^rd^ booster immunization in 30 and 100 µg UB-612 dose groups were evaluated in live virus CPE-based neutralization assays against different variants using group pooled sera. The UB-612 induced broadly NAb responses against D614G, Alpha (B.1.1.7), Beta (B.1.351), Gamma (P.1) and Delta (B.1.617.v2) VOCs. However, the titers against Gamma (P.1) and Beta (B.1.351) variants were reduced (Figure 4(A)).

VSV-based-pseudoviruses were generated to express individual S protein from WT strain, three VOCs: Alpha (B.1.1.7), Beta (B.1.351) and Gamma (P.1), and two VOIs: Epsilon (B.1.429) and Iota (B.1.526), to evaluate the breadth of neutralizing activities. Macaque sera from the 100 µg dose group, collected on Days 42 and 70 (two- and six-weeks post-dose 2) and Day 77 (one-week post-dose 3), neutralized all six viruses tested. The 3^rd^ dose booster immunization increased the neutralization activity against all six viruses (Figure 4(B)). Compared to WT or D614G strains, the neutralizing activities against Alpha, Gamma, Delta, and Epsilon variants were similar, but reduced against Iota and Beta variants by >50%. NAb titers peaked at two weeks after the 2^nd^ immunization (Day 42) and then started to wane on Day 70 before the 3^rd^ booster dose. After the 3^rd^ immunization, NAb titers against all viruses were significantly increased surpassing the peak titers after the 2^nd^ immunization.

Taken together, UB-612 vaccine induced broadly neutralizing antibodies against multiple SARS-CoV-2 variants in macaques.

### Immunogenicity and protection against SAR-CoV-2 challenge after two-dose UB-612 vaccine in cynomolgus macaques

#### Antibody responses

**Figure 5.**
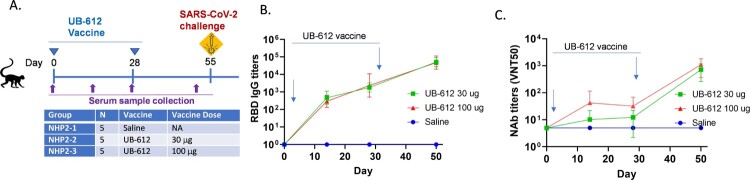
Antibody responses in UB-612 immunized cynomolgus macaques. (A) UB-612 vaccine and saline immunization groups, dosing, and challenge schedule. (B) Temporal RBD-specific antibody responses. (C) Temporal NAb responses against SARS-CoV-2 WA strain. The arrows indicate the time points of immunization. Each curve represents GMT ± SD.

In the 2^nd^ NHP study, cynomolgus macaques received 30 or 100 μg of UB-612 or saline on Days 0 and 28 (Figure 5(A)). After the 1^st^ immunization, both dose groups elicited RBD-specific IgG and NAb responses. The 2^nd^ immunization significantly boosted the RBD-specific IgG (Figure 5(B)) and NAb responses against the SARS-CoV-2 wild type WA strain in a live virus CPE-based neutralization assay (Figure 5(C)). The 100 μg dose group generated higher NAb titers than the 30 μg dose group.

**Figure 6.**
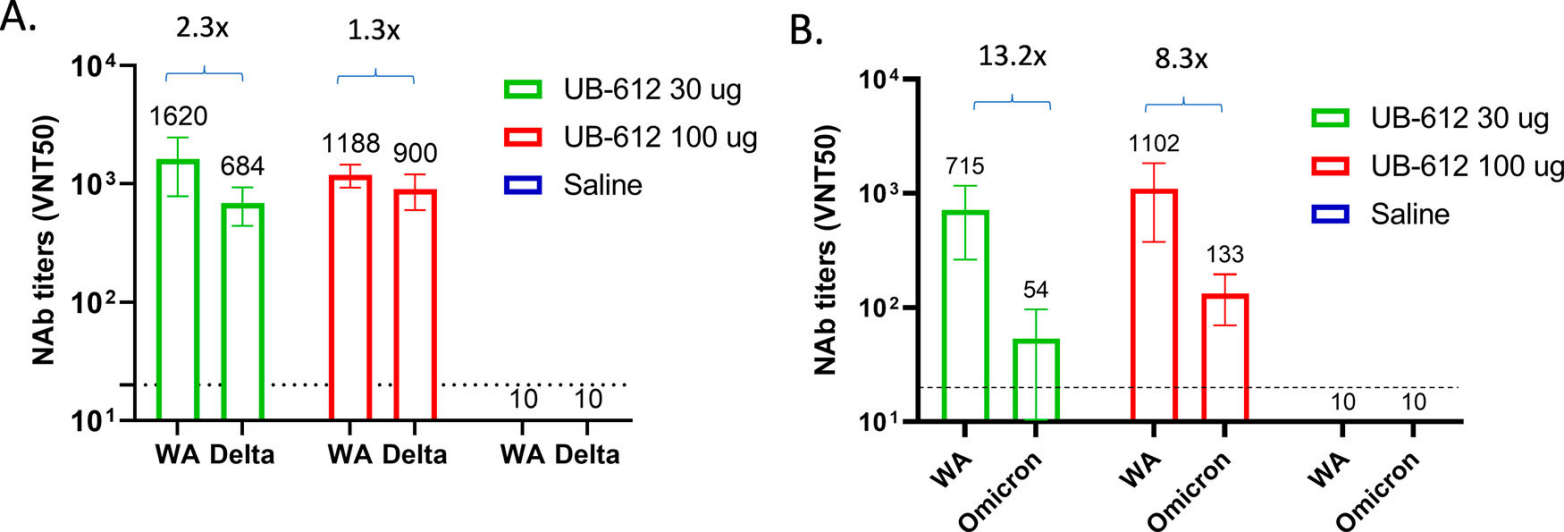
NAb titers against SARS-CoV-2 WA stain, Delta (A) and Omicron (B) VOCs in cynomolgus macaque sera collected 3 weeks post the 2^nd^ immunization. A. CPE NAb titers; B. Microneutralization NAb titers. The bar represents group GMT ± SD, with GMT value above.

We further evaluated NAb responses against live viruses using CPE-based assays to compare WA strain and Delta variant and microneutralization assays to compare WA strain and Omicron (BA.1) variant. Two-dose UB-612 also elicited NAb responses against Delta (Figure 6(A), Supplemental Figure S5B) and Omicron (Figure 6(B), Supplemental Figure S5C). However, the titers against both VOCs were decreased compared to WA strain. NAb titers against Delta and Omicron in 100 μg dose group decreased by 1.3- and 8.3-fold, respectively, which was less of a decrease than observed in 30 μg dose group (2.3-fold and 13.2-fold for Delta and Omicron BA.1, respectively).

#### T-cell responses

**Figure 7.**
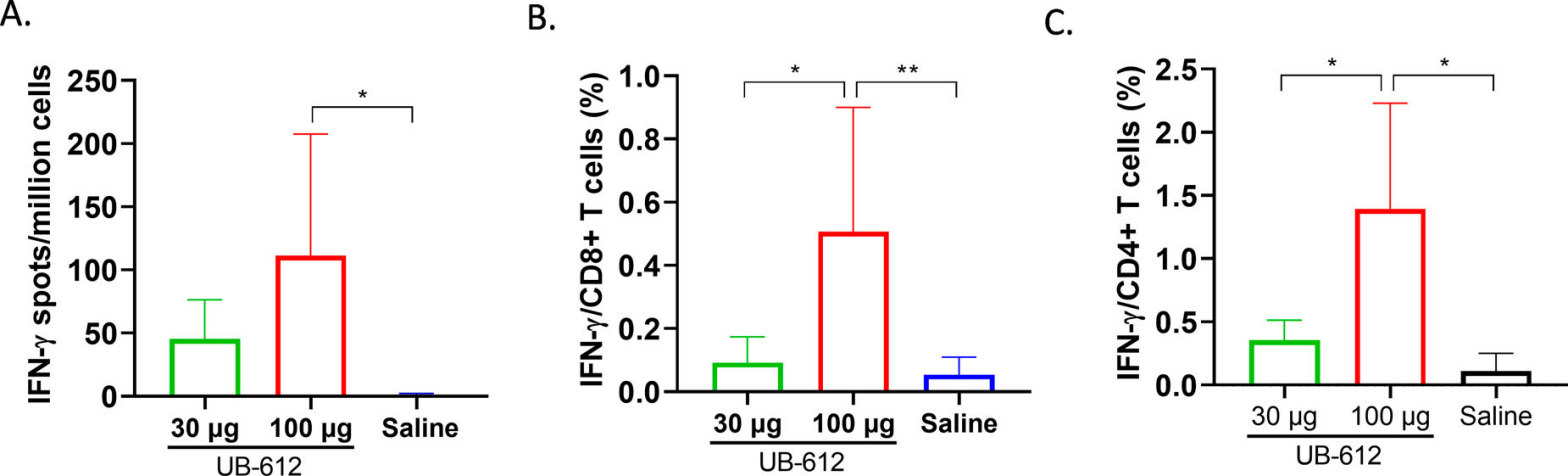
SARS-CoV-2 S2/M/N peptide specific IFN-γ T-cell responses in cynomolgus macaque PBMCs collected at 3 weeks post the 2^nd^ UB-612 immunization. ELISpot (A) and intracellular cytokine staining of CD8 (B) and CD4 (C) T-cells. The IFN-γ T-cell ELISpots, CD8 and CD4 responses are presented as GMT ± SD. Statistical significance between groups is indicated, *p < 0.05; **p < 0.01.

UB-612 vaccine peptide-specific T-cell responses were evaluated in PBMCs collected three weeks after the 2^nd^ immunization (Day 50) by IFN-γ and IL-4 T-cell ELISpots and intracellular cytokine staining (ICS). IFN-γ secreting spots were detected in both 30 and 100 μg dose groups (Figure 7(A)), but no IL-4 secreting spots were detected after stimulation with mixed S2/M/N peptides (data not shown). We also analyzed IFN-γ and IL-4 responses in CD8+ and CD4+ T-cells by ICS. IFN-γ production was detected in both CD8 + and CD4+ T-cells (Figure 7(B,C)), but IL-4 was not detected after stimulation with S2/M/N peptides, similar to the ELISpot results. T-cell responses were dose-dependent with 3∼5-fold increase in IFN-γ-secreting T-cells observed in ELISpot and ICS assays in PBMCs from 100 μg dose group compared to 30 μg dose group (Figure 7). No SARS-CoV-2 peptide-specific T-cell responses were detected in saline group, as expected.

#### Protection against SARS-CoV-2 WA strain challenge

**Figure 8.**
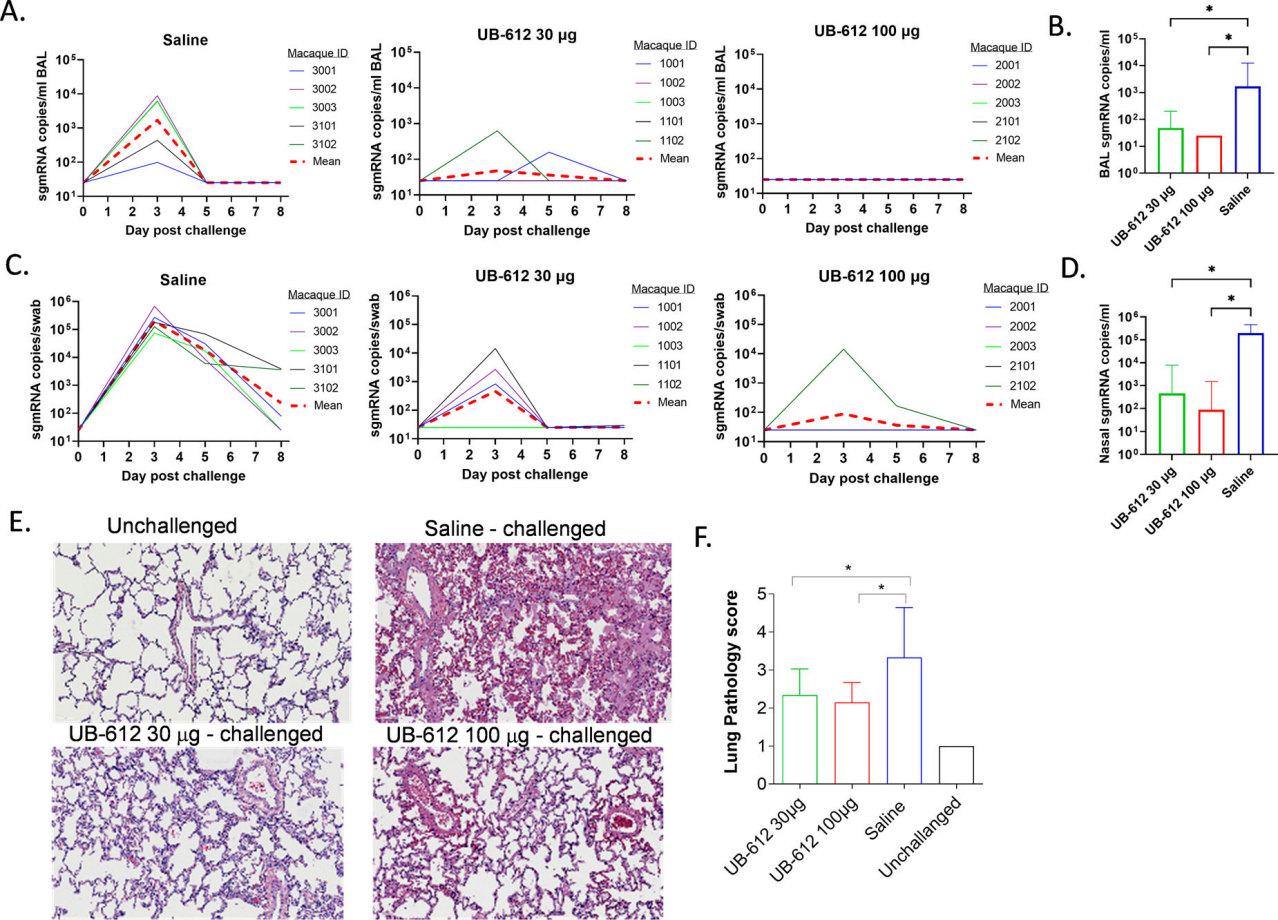
Protection against SARS-CoV-2 WA strain IT/IN challenge in cynomolgus macaques at 3 weeks post the 2^nd^ immunization. Animals were immunized with saline, 30 or 100 μg of UB-612 vaccine. The temporal viral sgmRNA levels detected in BAL (A) and nasal swabs (C) of individual animals. The solid curves represent individual macaques while the red dashed curves represent GMT. The peak sgmRNA levels detected in BAL (B) and nasal swab (D) samples of each group on Day 3 post-challenge. The peak viral loads are presented as GMT ± SD. (E) Examples of histopathology of lung samples collected on Day 8 post-challenge (magnitude x 40). (F) The lung pathology scores presented as GMT ± SD. Statistical significance is indicated in panels B, D and F for 30 or 100 μg UB-612 dose group compared to Saline group, *p < 0.05.

Cynomolgus macaques were challenged by combination of IT (4 × 10^4^ TCID_50_) and IN (4 × 10^4^ TCID_50_) infections with SARS-CoV-2 WA strain at three weeks after the 2^nd^ immunization (Day 55). After challenge, viral sgmRNA loads were measured in BAL (Figure 8(A)) and nasal swabs (Figure 8(C)) for eight days. While all saline immunized animals had high levels of viral sgmRNA in BAL, only 2 of 5 animals in UB-612 30 μg dose group were detected with lower levels of sgmRNA and none of 5 animals in UB-612 100 μg dose group had detectable sgmRNA. Similarly, in nasal swabs, only 2 of 5 animals in UB-612 30 μg dose group, and 1 of 5 animals in UB-612 100 μg dose group, had much lower levels of sgmRNA, while all saline immunized animals had high levels of sgmRNA (Figure 8(C)). Peak viral loads were measured in BAL (Figure 8(B)) and nasal swabs (Figure 8(D)) on Day 3 post-challenge and viral loads were also calculated as area-under-curve (AUC) from Day 0–8 (Supplemental Figure S6), with significant reductions in vaccine groups. UB-612 vaccination protected both upper and lower respiratory tracts of immunized macaques with significant viral load reductions compared to saline group.

On Day 8 post-challenge, lung tissues were collected from individual macaques for histopathological analyses. UB-612 vaccinated animals in both dose groups had significant reductions in inflammatory cell infiltration and lower lung pathology scores compared to saline group (Figure 8(E) and (F)).

Taken together, the virological and pathology evaluations in lungs demonstrated that UB-612 vaccination protected macaques against SARS-CoV-2 infection, reduced lung tissue pathology and disease progression after two-dose primary immunizations. The vaccine induced protections were dose dependent.

## Discussions

New generation COVID-19 vaccines, capable of eliciting potent and broad NAbs to prevent viral infection and inducing T-cell responses to clear the infected cells, would offer a more promising solution to control the pandemic. UB-612 is a next generation vaccine with an antigenic composition designed specifically to generate both humoral and cellular immunity. UB-612 is comprised of RBD to generate NAbs and memory B cell responses with broad reactivity to VOCs, including Beta, Delta, and Omicron [23], and conserved Th/CTL peptides from S, M and N proteins to generate T-cell responses across major VOCs, including Omicron.

Many vaccines against viral diseases, including COVID-19, protect through eliciting NAb responses [24, 25]. Falling neutralizing titers over time may also raise concerns about antibody-dependent enhancement (ADE) or vaccine associated enhanced respiratory disease (VAERD) upon exposure to circulating SARS-CoV-2 [26]. Safety risks associated with ADE or VAERD were previously described for SARS and MERS coronaviruses [27]. The ADE and VAERD responses may be caused by mechanisms unknown and dependent of the amino acids 597–603 sequence in S2 which have been implicated in ADE of SARS-CoV *in vitro* and in NHPs [28]. The RBD subunit antigen does not contain S2 subunit and no ADE or VAERD was observed in any animals vaccinated with UB-612 and challenged with SARS-CoV-2.

Considering the role of T-cells, a balanced Th/Th2 immunity is strongly believed to provide added protective immunities to the viral vaccines. The dominant IFN-γ and IL-2 T-cell responses in rats and macaques demonstrated that UB-612 vaccination can induce robust SARS-CoV-2 peptide-specific T-cell responses and confirmed Th1-prone cellular immune responses as previously reported [13]. These data also agree with previous observations showing that CpG-1018 and Alum combination with prefusion S-2P immunogen can induce strong and cross-neutralizing Th1 dominant immune response in mice to prevent COVID-19 [29], despite the amounts of CpG1 in all our studies were significantly lower. The CpG doses in our preclinical and clinical studies were 100s–1000s times lower than the CpG amounts present in several licensed human vaccines to date. For initial mouse immunizations, a 2-week interval was conducted. For monkey immunizations, as well as for our clinical studies, the 4-week interval was conducted to allow a longer interval time between the first and second immunizations, shown to influence the efficacy of COVID-19 vaccines [30].

While NHP models do not reproduce severe COVID-19 disease seen in humans, the virus can cause infection and illness in macaques, and the disease is generally mild, self-limiting and resolves within two weeks (19). Cynomolgus macaques immunized with two doses of UB-612 demonstrated strong immune responses and were protected against SARS-CoV-2 IN/IT challenge. Our results are consistent with previous studies which have shown that RBD-Fc or RBD vaccines induce high levels of NAbs in NHPs. A yeast-expressed RBD-based vaccine formulated with 3M-052-alum significantly reduced viral loads in respiratory tracts and lung inflammation in rhesus macaques in a SARS-CoV-2 challenge study, with NAbs peaking after the 3^rd^ vaccination [31].

An additional 3^rd^ dose booster immunization with the RBD may improve neutralizing antibodies in nonhuman primates [32]. The booster immunization concept was addressed in a rhesus macaque study testing three-dose vaccination. A 3^rd^ dose UB-612 homologous booster generated high titers of RBD-specific IgG and potent NAb responses. Furthermore, UB-612 induced potent NAbs against multiple VOCs in live virus and pseudovirus neutralization assays, including Alpha, Beta, Gamma, Delta, and Omicron BA.1. Similarly, in our Phase 1 clinical study conducted in Taiwan, a booster dose of UB-612 vaccine administered 7–9 months after primary vaccination increased NAb levels by 131-, 61- and 49-fold against the ancestral SARS-CoV-2, Omicron BA.1, and BA.2 variants, respectively [33]. Based on RBD-specific binding antibody responses from our Phase 1 and Phase 2 study participants, we have predicted 82% and ∼95% efficacy against symptomatic COVID-19 caused by the ancestral strain [33] although the efficacy of primary immunization with UB-612 has not been determined in large Phase 3 studies mainly due to difficulty in recruiting COVID-19 and COVID-19 vaccine naïve subjects.

UB-612 protein/peptide subunit vaccine has several key differentiators distinguishing it from the available COVID-19 vaccine options. (1) Longevity of NAbs: in clinical trials, UB-612 induced long lasting NAbs with a half-life of 187 days (post primary immunization), which was higher than, 40 days for an inactivated vaccine [34], 68 days reported for mRNA vaccines [35, 36], and 108–150 days observed in COVID-19 infected individuals [37, 38]. (2) Variant specific responses: a booster dose of UB-612 induced comparable levels of Omicron-specific neutralizing antibodies and RBD-specific IgG to those elicited by 3 dose Pfizer mRNA vaccine [33], indicating it could match the efficacy of mRNA boosters to combat Omicron and other VOCs. (3) Safety: UB-612 uses aluminum adjuvant (in use for childhood immunizations over 70 years) which demonstrated favorable safety profiles in Phase 1 and Phase 2 clinical studies in ∼ 4000 subjects, including adolescents and the elderly [13]. (4) High levels of antibody responses: UB-612 induced RBD-specific IgG and NAb responses were higher than those induced by inactivated vaccine in clinical trials [13, 33, 39]; (5) Broad T cell responses against conserved T cell epitopes in S2, M and N proteins. (6) Storage: UB-612 vaccine has targeted product attributes allowing for extended storage (24 months) and compatibility with existing cold-chain logistics.

In summary, UB-612 vaccine elicited high levels of neutralizing antibodies with breadth across multiple major SARS-CoV-2 variants including Alpha, Beta, Gamma, Delta and Omicron [33] and a Th1-prone immune response. UB-612 vaccine generated protective immunity against high dose SARS-CoV-2 respiratory challenges in mouse and NHP models, including a significant virus load reduction in both upper and lower respiratory tracts, pathological scores in lungs and disease progression. Our results also support the use of UB-612 as a booster for other COVID-19 vaccines and improve protection against current and emerging SARS-CoV-2 variants. Currently, UB-612 is being evaluated as a heterologous booster dose in a global pivotal Phase 3 clinical trial (ClinicalTrials.gov Identifier: NCT05293665). The goal of Phase 3 clinical trial, co-funded by the Coalition for Epidemic Preparedness Innovation (CEPI), is to determine whether UB-612 can serve as a safe next generation subunit booster vaccine to restore protective immunity against SARS-CoV-2 in subjects who completed primary immunization with the first-generation mRNA-, adenovirus-based- or inactivated vaccines.

## Supplementary Material

Supplemental Material

## Data Availability

All reasonable requests for data associated with this manuscript should be routed to the corresponding author.
