# Supplementary material for: A novel RBD-protein/peptide vaccine elicits broadly neutralizing antibodies and protects mice and macaques against SARS-CoV-2
Source: Emerg Microbes Infect. 2022 Nov 11;11(1):2724–34. doi: 10.1080/22221751.2022.2140608 (PMC9662039; doi:10.1080/22221751.2022.2140608)
Supplement: Supplemental Material [file TEMI_A_2140608_SM5253.pdf]

Supplemental Materials for

A Novel RBD-Protein/Peptide Vaccine Elicits Broadly Neutralizing Antibodies and Protects Mice and Macaques against SARS-CoV-2

Shixia Wang[1#], Chang Yi Wang[2,3,4#], Hui-Kai Kuo[2], Wen-Jiun Peng[2],Juin-Hua Huang[2], Be-Shen Kuo[3], Feng Lin[4], Yaw-Jen Liu[3], Zhi Liu[4], Huang-Ting Wu[2], Shuang Ding[4], Kou-Liang Hou[2], Jennifer Cheng[2], Ya-Ting Yang[2], Ming-Han Jiang[2], Min-Sheng Wang[2], Tony Chen[2], WeiGuo Xia[4], Ed Lin[4], Chung Ho Hung[4], Hui-Jung Chen[3], Zhonghao Shih[3], Yilin Lin[5], Valorie Ryan[1], Mei Mei Hu[1,3,4], D. Gray Heppner[1], Delphine C. Malherbe[6,8], Sivakumar Periasamy[6,8], Natalia Kuzmina[6,8], Chandru Subramani[6,8], Michael Hellerstein[1], Thomas P. Monath[1], Alexander Rumyantsev[1], Alexander Bukreyev[6,7,8], Farshad Guirakhoo[1*]

[1] Vaxxinity Inc., Dallas, TX, USA; [2] United Biomedical Inc., Asia, Taipei, Taiwan;

[3] United BioPharma, Hsin Chu, Taiwan; [4] United Biomedical Inc., Hauppauge, NY, USA; [5] Academia Sinica, Nangang,Taipei, Taiwan;

[6] Department of Pathology, [8] Department of Microbiology & Immunology, University of Texas Medical Branch, Galveston, TX, USA; [7] Galveston National Laboratory, Galveston, TX, USA

# Equal contributions

* Corresponding author

Email: farshad@vaxxinity.com

Supplemental Methods

Generation of CHO stable cell line for RBD-sFc protein expression and confirmation of binding activity

A stable high-expressing clone for RBD-sFc protein was isolated and a stable CHO expression cell line was generated. Briefly, the DNA sequence encoding RBD-sFc was synthesized and cloned into Freedom® pCHO 1.0 vector (Life Technology) to obtain the pCHO/RBD-sFc expression plasmid. A stable CHO cell line expressing RBD-sFc was generated through transfection of CHO cells followed by dihydrofolate reductase (DHFR) amplification. The CHO cell culture media were harvested, clarified by centrifugation and 0.22-μm filtration, and purified by Protein A chromatography. The qualities of the fusion protein were determined by SDS-PAGE and spectrophotometry. Peptide mapping, N- and C-terminal amino acid sequencing, and analysis of disulfide bonding and glycosylation confirmed that the expressed and purified protein conformed to the predicted characteristics. Size-exclusion chromatography (SEC), analytical ultracentrifugation, and capillary electrophoresis with sodium dodecyl sulfate experiments demonstrated that RBD-Fc exists in two major isoforms, RBD-sFc1 and RBD-sFc2, corresponding to N-linked and O-linked glycoforms of the protein. The binding activity of the vaccine was tested in an hACE2 ELISA and was demonstrated to bind hACE2 with a 50% effective concentration (EC_{50}) of 8.477 ng/mL, indicative of high affinity. The purified and characterized RBD-sFc protein was used for animal immunization studies.

Th/CTL epitope design

For Th/CTL epitope design, we employed the “Epitope Prediction and Analysis Tools”[1] to identify the desirable CTL T-cell epitopes. Th/CTL epitopes from highly conserved sequences derived from all three SARS-CoV-2 proteins (S, N and M) proteins were identified through an extensive literature search and epitope analysis [1,2]. Five peptides within these regions were selected for inclusion in the UB-612 immunogen (S2/M/N peptides): three S2 peptides (p5752, p5753 and p5755), one N peptide (p5754), and one M peptide (p5815). All peptides were chemically synthesized at UBIAsia (Taiwan).

In the UB-612 vaccine formulation for all doses, the ratio of RBD-sFc protein and Th/CTL peptides were 88% vs 12% concentration, respectively. For example for the 100 μg dose we used 88 μg RBD-sFc (88%) +12 μg (12%) of Th/CTL peptides (p5752 + p5753 + p5754 + p5755 + p5815 + UBITh1a)= 1:1:1:1:1:1 w/w ratio], and for the 10 μg dose group we used 8.8 μg RBD-sFc (88%) + 1.2 μg (12%) of Th/CTL (p5752 + p5753 + p5754 + p5755 + p5815 + UBITh1a)= 1:1:1:1:1:1 weight/weight ratio].

The T cell peptides were further analyzed using Immune Epitope Database & Analysis Resources (https://www.iedb.org/) to evaluate the potential MHC-I and MHC-II HLA coverage for different populations. These peptides showed a broad coverage of human MHC-I and MHC-II alleles including HLA-A*01:01, HLA-A*02:01, HLA-A*02:03, HLA-A*31:01, HLA-A*33:03, HLA-B*13.01, HLA-B*15.02, HLA-B*58.01, HLA-DRB1*01:01, HLA-DRB1*07:01, HLA-DRB1*12:02, HLA-DQB1*06:02 in different populations including South and East Asian, Caucasian, and African populations.

Rat immunogenicity study

Male Sprague Dawley rats at 8-10 weeks of age (300-350 gram of body wight) were purchased from BioLASCO Taiwan Co., Ltd. Two groups of rats were vaccinated intramuscularly (IM; 0.5mL) at Weeks 0 and 2 with two different doses (10 μg and 30 μg) of UB-612 vaccine.

The immune sera from rats (n=3 animals/group) were collected at Weeks 0, 2, 3 and 4 to evaluate RBD-specific IgG and NAb responses. The rat splenocytes were collected at Week 4 to evaluate cell-mediated immune responses. All procedures on animals were performed in accordance with the regulations and guidelines reviewed and approved by the institutional animal care and use committee (IACUC) at United Biomedical Inc Asia (UBIAsia, Taipei, Taiwan).

ELISA to detect RBD-specific antibody in mice, rats, and macaques

ELISA was performed to evaluate RBD-specific antibody responses in mice, rats, and macaques. Briefly, microtiter 96-well ELISA plates were coated with 100 µL/well of RBD-His protein (UBP, Taiwan) at 2 µg/mL overnight at room temperature (RT).

After blocking, 100 µL/well of serially diluted serum samples in replicates was added and incubated at 37°C for 1 hr. The HRP-conjugated-Protein A/G (United BioPharma, Hsin Chu, Taiwan), or HRP-conjugated goat-anti-monkey IgG (ACROBiosystems, Newark, DE) as detection antibodies (100 µL/well) was incubated for 30 minutes at 37˚C. Finally, TMB (3,3',5,5'-tetramethylbenzidine, 100 µL/well) was incubated at 37˚C for 15 min and then stopped with 1M H_2SO_4. Washes were performed between steps. The absorbance at 450 nm was measured with

an ELISA plate reader. The RBD-specific antibody titers were further determined as the reciprocal of the highest serum dilution.

ELISA to measure inhibition of RBD and human ACE2 binding

Microtiter 96-well ELISA plates were coated with 2 µg/mL of ACE2-ECD-Fc protein in 0.1 M sodium carbonate buffer (pH 9.6) overnight (16 to 18 hrs) at 4°C. After blocking, properly diluted immune serum samples or a positive control antibody mixed with HRP-conjugated-RBD protein were incubated for 30 min at room temperature. Finally, 100 µL of TMB (3,3',5,5'-tetramethylbenzidine) was added into each well and incubated at 37°C for 15 min in the dark, and the reaction was stopped with 1M H_2SO_4, 1.0 M stop solution. Washes were performed between steps. The absorbance at 450 nm was measured with SpectraMax® M2e ELISA plate reader (Molecular Device, CA). RBD-hACE2 binding inhibition titers were determined using UBI® ELISA Titer Calculation Program as the reciprocal of the highest serum dilution.

SARS-CoV-2 live virus microneutralization assay

Vero-E6 cells were expanded, and their concentrations were adjusted to 1.5×10^5 viable cells/mL in culture medium (Dulbecco's Modified Eagle Medium (DMEM) containing 10% FBS). The 96-well microtiter plates were seeded with 1.5×10^4 cells/100 µL/well. The plates were incubated at 37°C in a CO_2 incubator overnight. The next day, serum samples from vaccinated mice, rats and macaques were diluted (1:5) starting with 72 µL of serum sample + 288 µL of dilution medium (DMEM, containing 5% FBS) to yield the first dilution. Then, 7x 2-fold serial dilutions were made with dilution medium (dilution points were adjusted according to the characteristics of the sample). The challenge virus (SARS-CoV-2-TCDC#4, a Taiwanese strain) was prepared at 100 $TCID_{50}$ in 50 µL of culture medium, incubated with 50 µL volume of each serum dilution (50 µL) (in triplicate) for 1 hr at 37°C, before adding to Vero-E6 cells in triplicate. Medium only was co-incubated with an equal volume of 100 $TCID_{50}$ of the viruses for 1 hr at 37°C and

used as 100% infected control. The plates were incubated at 37°C in a CO_2 incubator for 4 days. Cells were then fixed overnight with 100 µL of 10% formaldehyde prepared in phosphate buffered saline, pH 7.0-7.4, added into each well. The next day formaldehyde solution was discarded by inverting the plate, and 100 µL of crystal 0.5% violet staining solution was added into each well and incubated at room temperature for 1 hr. The infection rate was quantified by ELISA reader and image analysis. The infection rate of medium only at a challenge dose of 100 $TCID_{50}$ virus was set at 100%, and each serum dilution with greater than 50% infection was scored as infected. The 50% protective titer was determined by the Reed and Muench method [3,4]. The examples of live virus microneutralization titration curves are presented in Supplemental Figure S5-C.

SARS-CoV-2 live virus CPE neutralization assay

NAb titers against SARS-CoV-2 wild type viruses and D614G, Alpha B.1.1.7, Gamma P.1, Beta B.1.351, and Delta B.1.617.2 variants, were determined using the CPE assay conducted in a BSL-3 laboratory at the Viral and Rickettsial Disease Laboratory, State of California Department of Public Health, United States. NAb titers were measured by CPE-based live virus neutralization assay using cells challenged with SARS-CoV-2 variants in in vitro microneutralization assay. Vero-81 were cultured with MEM supplemented with 1x penicillin-streptomycin (Gibco) and glutamine (Gibco) and 5% Fetal calf serum (Hyclone). Determination of SARS-CoV-2 virus specific neutralization titer was by measuring the NAb titer against the viruses based on the principle of 50% virus neutralizing titer (VNT_{50}) (≥50% reduction of virus-induced cytopathic effects). VNT_{50} of a serum was defined as the reciprocal of the highest serum dilution at which 50% reduction in cytopathic effects occurred. The examples of CPE-based neutralizing antibody titration curves are presented in Supplemental Figure S5-B.

Generation of SARS-CoV-2 pseudoviruses

Site-directed mutagenesis was used to generate the VOC plasmids by changing nucleotides from the Wuhan-Hu-1 reference strain.

A pseudovirus luciferase assay was performed to evaluate NAb titers against wild-type Wuhan-HU-1 (WT) and VOCs (Alpha, Beta, Delta and Gamma). To produce SARS-CoV-2 pseudoviruses, a plasmid expressing C-terminal truncated wild-type Wuhan-Hu-1 strain SARS-CoV-2 spike protein (pcDNA3.1-nCoV-SΔ18) was co-transfected into HEK-293T/17 cells with packaging and reporter plasmids (pCMVΔ8.91, and pLAS2w.FLuc.Ppuro, respectively) (BioTReC, Academia Sinica), using TransIT-LT1 transfection reagent (Mirus Bio). At 72 hrs post-transfection, cell debris was removed by centrifugation at 4,000 x g for 10 min, and supernatants were collected, filtered (0.45 μm, Pall Corporation) and frozen at −80°C until use.

Pseudovirus neutralization assay

HEK-293-hACE2 cells ($1x10^4$ cells per well) were seeded in 96-well white isoplates and incubated overnight. Tested sera were heated at 56°C for 30 min to inactivate complement and were then diluted in medium (DMEM supplemented with 1% FBS and 100 U/ml Penicillin/Streptomycin). Two-fold serial dilutions were carried out for a total of 8 dilutions. Twenty-five microliters of diluted sera were mixed with an equal volume of pseudovirus (1,000 TU) and incubated at 37°C for 1 hour before adding to the plates with cells. After a 1-hour incubation, 50 μL virus-sera mixture was added to the plates (containing 50 μL of DMEM culture medium per well). After an additional 16-hour incubation, the culture medium was replaced with 50 μL of fresh medium (DMEM supplemented with 10% FBS and 100 U/ml

Penicillin/Streptomycin). Cells were lysed at 72-hour post-infection and relative light units (RLU) was measured using Bright-GloTM Luciferase Assay System (Promega). Luciferase activity was detected by the Tecan i-control (Infinite 500). The percentage inhibition was calculated as the ratio of RLU reduction in the presence of diluted serum to the RLU value of virus only control: (RLU Control - RLU Serum) / RLU Control. The 50% protective titer (VNT_{50} titer) was determined by the Reed and Muench method [3,4]. The examples of pseudovirus titration curves are presented in Supplemental Figure S5-A.

Rat and macaque T-cell ELISpot

For evaluation of peptide-specific IFN-γ and IL-4 T-cell responses by UB-612 vaccine, ELISpot assays were performed for rats and cynomolgus macaques.

Splenocytes isolated from rats at 2 weeks after the 2nd immunization were used to evaluate the peptide-specific IFN-γ and IL-4 T-cell responses. Splenocytes were then collected at Week 4 and restimulated *in vitro* with the individual S2 peptides (p5752, p5753 and p5755), N peptide (p5754), and M peptide (p5815), or pooled S2+N+Mpeptides. The rat T-cell ELISpot assays were performed using the Rat IFN-γ ELISpot^{PLUS} kit (MABTECH, Nacka Strand, Sweden), Rat IL-4 T-cell ELISpot kit (U-CyTech, Utrecht, Netherlands) and Rat IL-2 ELISpot Kit (R&D Systems, Minneapolis, MN). ELISpot plates precoated with capture antibody were blocked with LCM for at least 30 min at RT. 250,000 rat splenocytes were plated into each well and stimulated with individual Th/CTL peptides or a peptide pool for 18-24 hrs at 37°C. Cells were stimulated with a final concentration of 1 μg of each peptide per well in LCM.

PBMCs collected in macaque at 3 weeks after the 2nd immunization were used to measure the peptide-specific IFN-γ and IL-4 T-cell responses. After stimulation with pooled

S2+N+Mpeptides for 48 hours, IFN-γ and IL-4 secreting cells were detected using the ImmunoSpot human IFN-γ/IL-4 double color ELISpot kit. Cytokine detection and spot developing procedures were performed based on the manufacturer's instructions. Spots were scanned and quantified by AID iSpot reader. Spot-forming units (SFU) per million cells was calculated by subtracting the negative control wells.

Virus $TCID_{50}$ detection in mouse lung

Five days post IN challenge, AAV6/CB-hACE2 mouse lung tissues were collected, weighed, and homogenized, and supernatant was harvested for live virus titration. Briefly, Vero-E6 cells were inoculated with serially diluted samples in quadruplicate wells in DMEM supplemented with 1% FBS and penicillin/streptomycin. CPEs were observed for 4 days. $TCID_{50}$ was determined as the amount of virus causing CPE in 50% inoculated wells. Virus titers were expressed as $TCID_{50}$/mL.

Viral sgmRNA detection by RT-PCR

The levels of N gene sgmRNA were assessed by RT-PCR, using the set of primers (SG-N-F: CGATCTCTTGTAGATCTGTTCTC, SG-N-R: GGTGAACCAAGACGCAGTAT and Probe: FAM- TAACCAGAATGGAGAACGCAGTGGG -BHQ). A plasmid containing a portion of the N gene messenger RNA served as the control and semi-quantification standard. The cycling conditions were performed with a one-step PCR protocol: 48°C for 30 minutes, 95°C for 10 minutes followed by 40 cycles of 95°C for 15 2nds, and 1 minute at 55°C. Data were collected and analyzed by Applied Biosystems 7500 Real-Time PCR System (ThermoFisher). The number of copies of RNA per mL of BAL or nasal swab samples was calculated by extrapolation

from the standard curve and multiplying by the reciprocal of 0.2 mL extraction volume to give a practical range of 50 to 5 x 10^7 RNA copies per swab or mL BAL fluid.

Intracellular cytokine staining

Cynomolgus macaque PBMCs were cultured and stimulated in vitro with mixed S2/M/N peptides. Briefly, PBMCs in RPMI complete media were plated in 96-well plates (10^6 cells/well in 200 μL) and incubated with 1x Brefeldin A and GolgiStop (BioLegend). The cells were stimulated with mixed S2/N/M peptides (5 μg/mL) for 5 hours. Then, the cells were surface stained with Live/Dead Aqua, CD3-APC/Fire750 (clone SK7), CD4-PerCP/Cy5 (clone OKT4) and CD8-BV605 (clone SK1) antibodies for 30 min. After washing with PBS/BSA, cells were fixed/permeabilized using Intracellular Fixation & Permeabilization Buffer (BioLegend) and stained with a mixture of anti-IFN-γ-APC (clone B27) and anti-IL-4-PE (MP4-25D2) antibodies for 30 min at 4°C in the dark. Cells were resuspended in PBS and analyzed on an LSR Fortessa flow cytometer (BD Biosciences). Cell type and intracellular cytokine expression frequencies were calculated using FlowJo software (v10.8.0_CL).

Lung histopathology

At the end of mouse and macaque challenge studies, animals were euthanized, and lung tissues were collected, trimmed, processed, embedded, sectioned, and stained with Hematoxylin and Eosin. Histopathology was examined under the microscope and histopathology scores were determined.

To score the lung histopathology, lung sections were divided into 9 equal square areas using a 3×3 grid. Scores from each of the 9 areas were averaged. The scoring system was as follows: 0, Normal, no significant finding; 1, Minor inflammation with slight thickening of alveolar

septa and sparse monocyte infiltration; 2, Apparent inflammation, alveolus septa thickening with more interstitial mononuclear inflammatory infiltration; 3, Diffuse alveolar damage (DAD), with alveolus septa thickening, and increased infiltration of inflammatory cells; 4, DAD, with extensive exudation and septa thickening, shrinking of alveoli, restricted fusion of the thick septa, obvious septal hemorrhage and more cell infiltration in alveolar cavities; 5, DAD, with massive cell filtration in alveolar cavities and alveoli shrinking, sheets of septal fusion, and hyaline membranes lining the alveolar walls.

Supplemental Results

Rat immunogenicity studies

The immunogenicity of the UB-612 vaccine was first tested in Sprague-Dawley rats, to evaluate the antibody and T-cell immune response. The rats received either 10 µg or 30 µg of UB-612, which elicited similarly high levels of antibody responses after two immunizations at Weeks 0 and 2. The RBD-specific binding antibody geometric titers were 1:2,084 or 1:2,055 after the first immunization and boosted to 1:74,473 or 1:69,024 after the 2nd immunization in 10 µg or 30 µg dose group, respectively (**Supplemental Figure S2A**). The RBD:hACE2 binding blocking antibody geometric titers were 1:523.5 or 1:1724.7 in 10 µg or 30 µg dose group, respectively, after the 2nd immunization (**Supplemental Figure S2B**). The NAb titers against a wild type live virus SARS-CoV-2 (hCoV-19/Taiwan/4/2020, original Wuhan-like virus) expressed as virus neutralizing titers 50% (VNT_{50}) were $>10^4$ (geometric mean titers) in both dose groups (**Supplemental Figure S2C**) after the 2nd immunization.

To assess T-cell response induced by UB-612 vaccine, the rat splenocytes were collected after the 2nd immunization to evaluate the IFN-γ, IL-2 and IL-4 T-cell responses by ELISpots. After the stimulation with the individual or pooled peptides, high levels of IFN-γ and IL-2 (Th1 cytokine) responses were detected against individual S2 peptides (p5752, p5753 and p5755), N peptide (p5754) and M peptide (p5815), and pooled peptides, but IL-4 (Th2 cytokine) responses were very low (**Supplemental Figure S3**). These results demonstrated that UB-612 vaccine is highly immunogenic and induces a Th1-prone cellular immune response, as supported by the high ratio of IFN-γ/IL-4 or IL-2/IL-4.

Supplemental Reference

1. Grifoni A, Sidney J, Zhang Y, et al. A Sequence Homology and Bioinformatic Approach Can Predict Candidate Targets for Immune Responses to SARS-CoV-2. Cell Host and Microbe 2020; 27:671-680.e2.
2. Ferretti AP, Kula T, Wang Y, et al. Unbiased Screens Show CD8+ T Cells of COVID-19 Patients Recognize Shared Epitopes in SARS-CoV-2 that Largely Reside outside the spike Protein. Immunity 2020; 53:1095-1107.e3.
3. Reed LJ and Muench H. A simple method of estimating fifty-percent endpoints. Am J Hyg. 1938; 27:493–497.
4. Lei S, Yang J, Hu J, and Sun X. On the Calculation of TCID50 for Quantitation of Virus Infectivity. Virol Sin. 2021; 36: 141–144.

Supplemental Figures

A

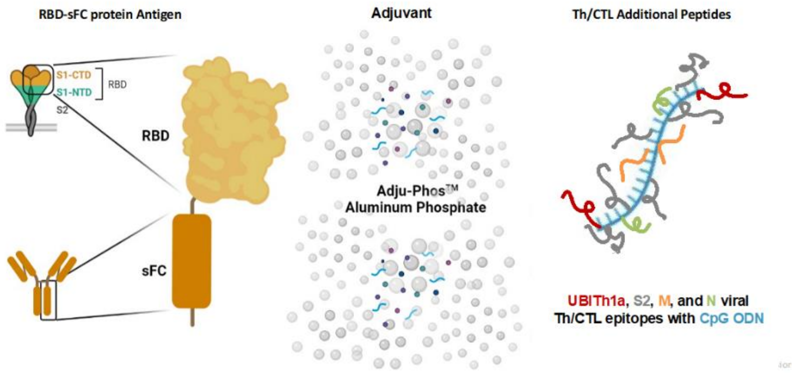

B

```
1   NITNLCPFGE VFNATRFASV YAWNRKRISN CVADYSVLYN SASFSTFKCY GVSPTKLNDL CFTNVYADSF
71  VIRGDEVRQI APGQTGKIAD YNYKLPDDFT GCVIAWNSNN LDSKVGGNYN YLYRLFRKSN LKPFERDIST
141 EIYQAGSTPC NGVEGFNCYF PLQSYGFQPT NGVGYQPYRV VVLSFELLHA PATVCGPKKS EPKSSDKTHT
211 SPPSPAPELL GGPSVFLFPP KPKDTLMISR TPEVTCVVVD VSHEDPEVKF NWYVDGVEVH NAKTKPREEQ
281 YHSTYRVVSV LTVLHQDWLN GKEYKCKVSN KALPAPIEKT ISKAKGQPRE PQVYTLPPSR DELTKNQVSL
351 TCLVKGFYPS DIAVEWESNG QPENNYKTTP PVLDSDGSFF LYSKLTVDKS RWQQGNVFSC SVMHEALHNH
421 YTQKSLSLSP G
```

N: N-linked glycosylation sites S: O-linked glycan site

Supplemental Figure S1. Components of the UB-612 vaccine.

(A) UB-612 vaccine formulation diagram. UB-612 vaccine contains an RBD-sFc fusion protein to elicit B cell responses, plus five synthetic Th/CTL peptides for class I and II MHC molecules derived from SARS-CoV2 S2, M, and N proteins and the UBITh1a peptide (a proprietary T helper peptide). The peptides stabilized in a complex with CpG and RBD-sFC subunit protein are adsorbed on Adju-Phos adjuvant to constitute the UB-612 vaccine final product.

(B) Sequence of RBD-sFc. RBD-sFc protein is a glycoprotein consisting of one N-

linked glycan (Asn13) and two O-linked glycans (Ser211 and Ser224). Light blue shading indicates the RBD of SARS-CoV-2 and no shading indicates the sFc fragment of an IgG1. The substitution of His297 for Asn297 (EU-index numbering) in single-chain Fc, His282 in RBD-sFc, is indicated by underline. RBD-sFc protein contains 431 amino acid residues, including 12 cysteine residues forming 6 pairs of disulfide bonds (Cys6-Cys31, Cys49-Cys102, Cys61-Cys195, Cys150-Cys158, Cys246-Cys306 and Cys352- Cys410), which are shown as orange lines.

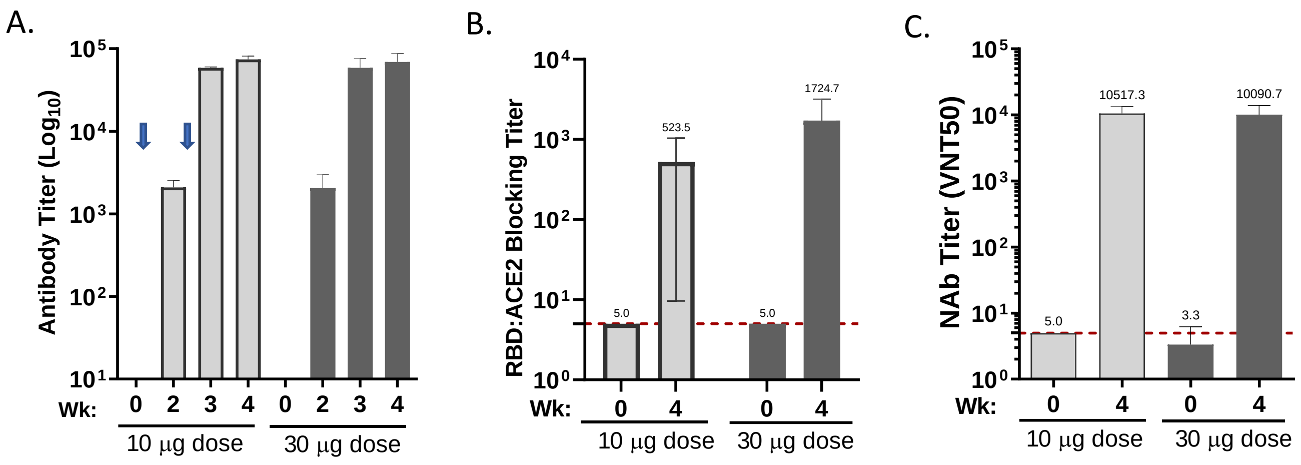

Supplemental Figure S2. RBD- binding and NAb responses of rats.

(A) S1-RBD specific antibody titers at 0-, 2-, 3-, and 4-weeks after UB-612 immunization at Week 0 and 2, measured by ELISA. The arrows indicate the immunization time points at Week 0 and 2. (B) RBD:hACE binding blocking antibodies measured in sera collected at week 4 (2 weeks after the 2nd immunization). (C) Neutralizing antibodies measured in sera collected at week 4 (2 weeks after the 2nd immunization). Neutralization titers expressed as VNT_{50}.

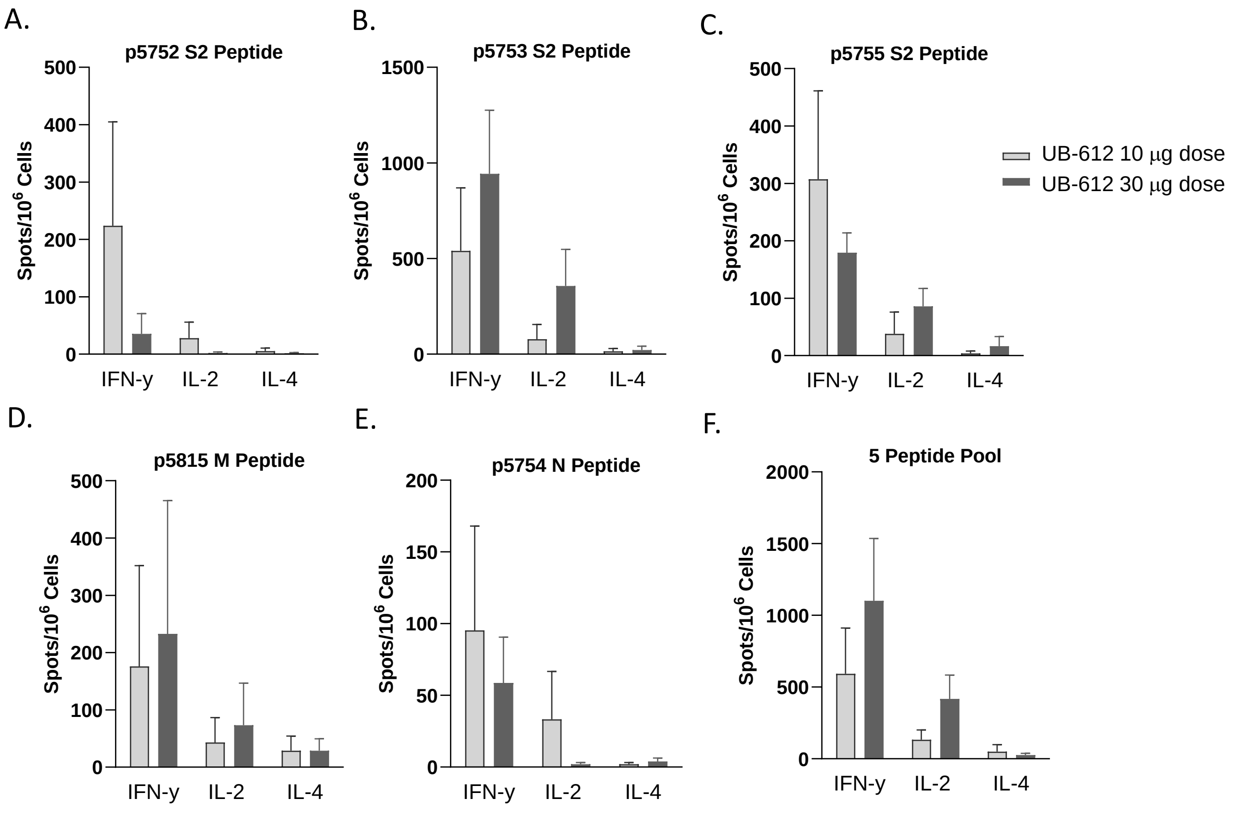

Supplemental Figure S3. S2/M/N peptide specific T-cell responses 2 weeks after the 2nd immunization.

IFN-γ, IL-2 and IL-4 producing cells from splenocytes were detected by T-cell ELISpot against S2 peptides (p5752, p5753 and p5755), N peptide (p5754), M peptide (p5815). The number of cytokine-secreting cells (spots) per million cells was calculated by subtracting the negative control wells. Bars represent the mean of each group with standard deviation. The light and dark grey bars represent the 10 μg and 30 μg group, respectively.

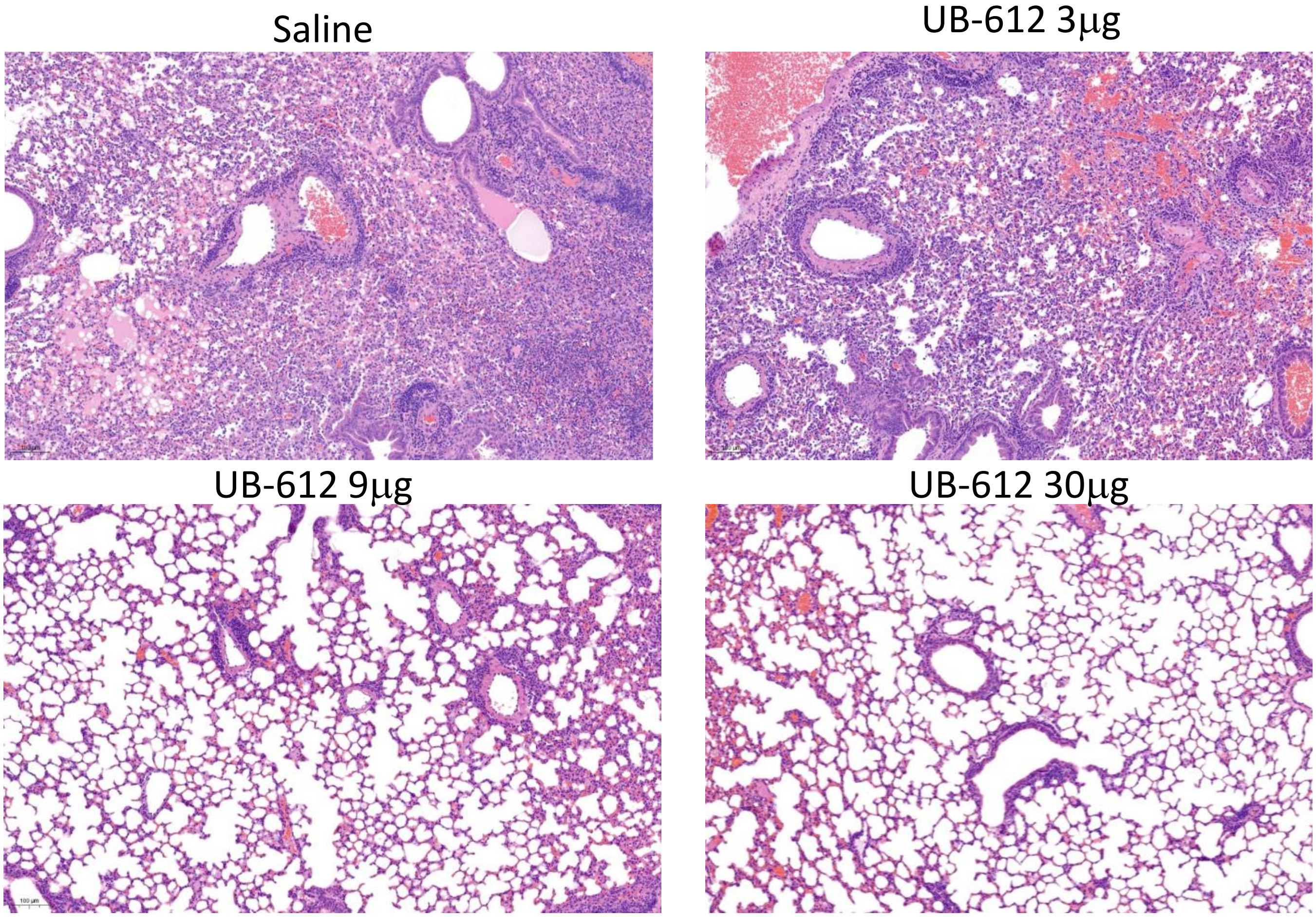

Supplemental Figure S4. Examples of histopathology of mouse lung samples. The lung samples were collected on Day 5 post intranasal challenge with SARS-CoV-2 in mice received 2 times of immunizations with Saline, UB-612 vaccine at 3, 9 or 30 μg dose. The histopathology microscopy example pictures are under magnitude x 40.

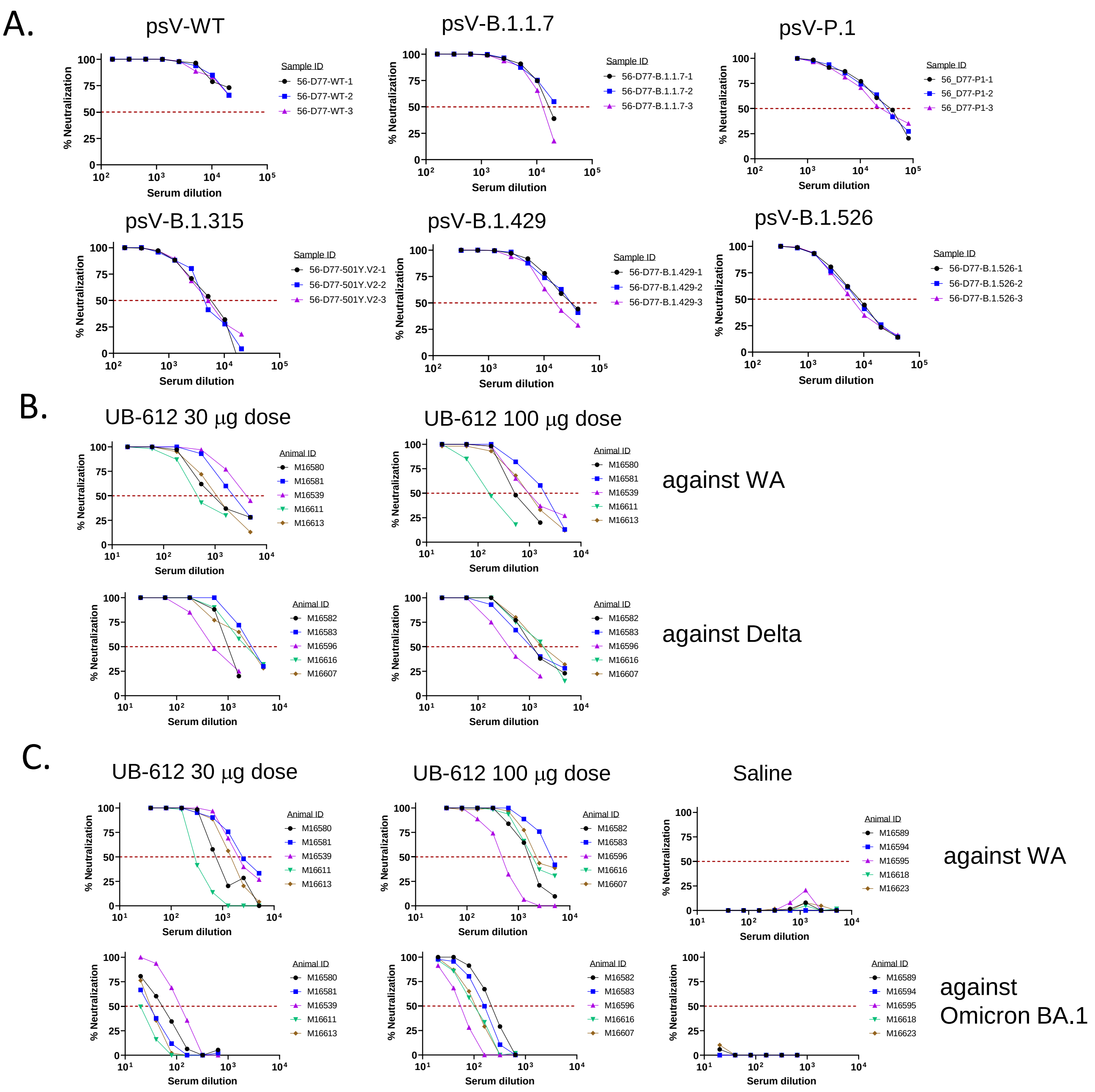

Supplemental Figure S5. Representative neutralizing antibody titration curve examples. A. Neutralizing antibody titration curves against VSV-based pseudoviruses expressing the spike protein from SARS-CoV-2 ancestral WT strain and multiple variants (B.1.1.7, P.1, B.1.315, B.1.429 and B.1.526) are as indicated in each panel. The serum samples used in these assays

were from the UB-612 100 μg dose group at 1 week post the third boost immunization in rrhesus macaque study. B. Neutralization titration curves against SARS-CoV-2 WA strain and Delta VOC live viruses are as indicated in CPE-based assays. The serum samples used in these assays were from the UB-612 30 and 100 μg dose groups at 2 weeks post the second immunization in cynomolgus macaque study as indicated. C. Neutralization titration curves against SARS-CoV-2 WA strain and Omicron VOC BA.1 live viruses are as indicated in microneutralization assays. The serum samples used in these assays were from the UB-612 30 and 100 μg dose groups and the Saline group at 2 weeks post the second immunization in Cynomolgus macaque study as indicated.

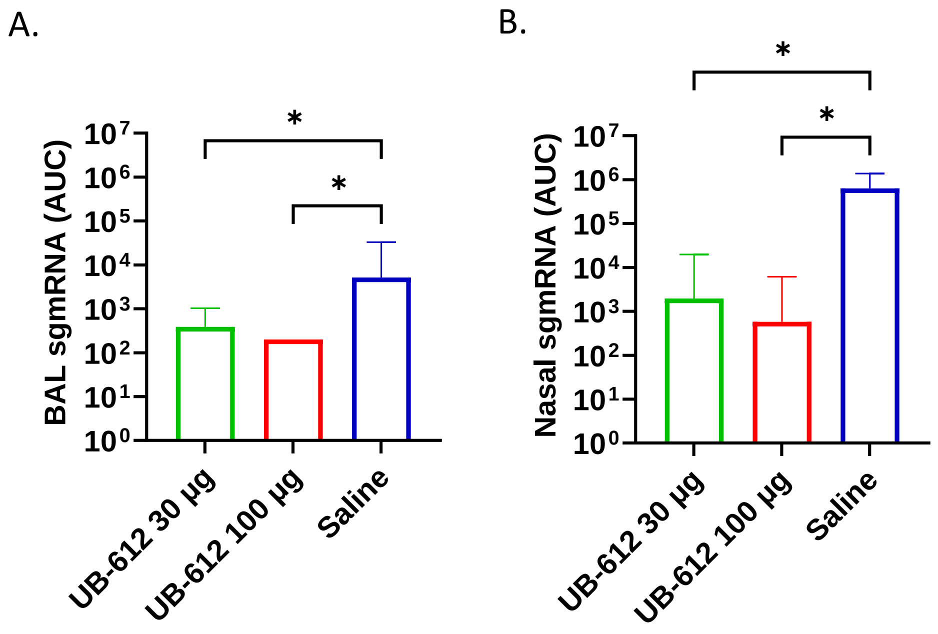

Supplemental Figure S6. Protection against SARS-CoV-2 wild type WA strain IT and IN challenge in cynomolgus macaques three weeks post the 2nd immunization with UB-612. Animals were immunized with saline, 30 or 100 µg of UB-612 vaccine. The sgmRNA levels detected in BAL (A) and nasal swab (B) samples of each group were calculated as the area-under-curve (AUC) from Day 0 to Day 8 post-challenge. The viral loads are presented as geometric mean of each group with standard deviation. The "*" indicates the statistical significance, $p<0.05$.
